# Output of MEMS Piezoelectric Energy Harvester of Double-Clamped Beams with Different Width Shapes

**DOI:** 10.3390/ma13102330

**Published:** 2020-05-19

**Authors:** Lei Jin, Shiqiao Gao, Xiyang Zhang, Qinghe Wu

**Affiliations:** School of Mechatronic Engineering, Beijing Institute of Technology, Beijing 100081, China; jinlei@bit.edu.cn (L.J.); zhangxiyang2012@163.com (X.Z.); wuqh_123@163.com (Q.W.)

**Keywords:** double-clamped, width shapes, piezoelectric energy harvester, electrodes pair, MEMS structure

## Abstract

For a microelectromechanical system (MEMS) piezoelectric energy harvester consisting of double-clamped beams, the effects of both beam shape and electrode arrangement on the voltage outputs are analyzed. For two kinds of harvester structures including millimeter-scale and micro-scale, and different shapes including rectangular, segmentally trapezoidal and concave parabolic are taken into account. Corresponding electric outputs are calculated and tested. Their results are in good agreement with each other. The experimental results validate the theoretical analysis.

## 1. Introduction

In general, a piezoelectric energy harvester is composed of beam structures. Because the bending curvature of beam is not a constant along the length direction whether for a cantilever beam or a double-clamped beam, the strain distributions is not uniform. Therefor the efficiency of energy harvesting is not good enough. To improve the output efficiency, in recent years, much effort has been put into the aspect of the optimization of the shapes. Based on studies of the trapezoidal shapes [1,2,3,4], arrayed trapezoidal beams [5], reversed trapezoidal beams [6], triangular shapes [7], and optimized shapes [8,9], Jin, Gao et al. [10] explained the shape effects of cantilever beams on electric outputs by an analytical method. At the same time, non-linear trapezoidal shapes of cantilever beams [11] and composite cantilever beams [12] were also investigated.

In addition to the cantilever beam, the other typical forms are the double-clamped beam structures. These structures are usually used in hybrid piezoelectric-electromagnetic energy harvesters [13,14,15,16,17]. They have better stability and symmetry than the cantilever beam so as to be more conducive to the stable movement of the magnet mass. By contrast with the unidirectional curvature of cantilever beam, there are different directional curvatures for a double-clamped beam. Optimization concerns not only the beam shapes but also the arrangement of the electrodes. However, so far research on the shapes of the beam and the arrangement of the electrode pair, especially for a microelectromechanical system (MEMS) energy harvester, is still rare. Therefore, it is necessary to study the optimal shapes of the beam and the arrangements of the electrode pair for a harvester with a double-clamped beam.

In this paper, a dynamic analytical method is presented to analyze the piezoelectric energy harvester consisting of double-clamped beams. The effects of both beam shape and the electrode arrangement for the MEMS energy harvester are studied analytically. At the same time, corresponding experiments are conducted both for millimeter-scale and micro-scale structures.

## 2. Governing Equations of the Piezoelectric Energy Harvester

**Figure 1:**
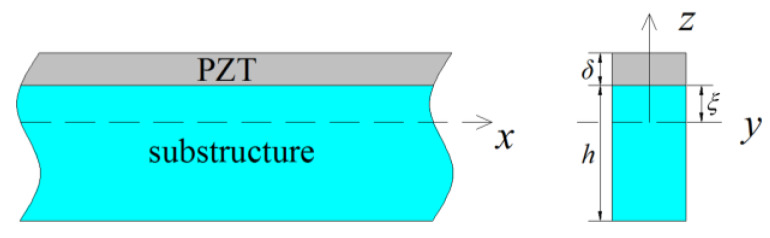
A composite beam.

For a bending beam, the strain $\varepsilon_{1}$ along the length direction can be written by $\frac{z}{\rho \left(x\right)}$, where $\frac{1}{\rho \left(x\right)}=\frac{\partial^{2}w}{\partial x^{2}}$ is the bending curvature of the beam. According to the direct effect and the converse effect described by Meeker [18] (IEEE Standard on Piezoelectricity), the constitutive equations of composite beam consisting of a base structure and a piezoelectric layer shown as in Figure 1 can be written by:

(1) $$\{\begin{array}{cc} T_{1}=Y_{p}\frac{z}{\rho \left(x\right)}-e_{31}E_{3} & \xi \leq z\leq \;\xi +\delta \\ T_{1}=Y_{s}\frac{z}{\rho \left(x\right)} & -\left(h-\xi \right)\leq z<\xi \\ D_{3}=e_{31}\frac{z}{\rho \left(x\right)}+\varepsilon_{33}E_{3} & \xi \leq z\leq \;\xi +\delta \end{array}$$

where x is the coordinate in length direction of composite beam, z is the coordinate in thickness direction, $T_{1}$ is the stress in the length direction x, $E_{3}$ and $D_{3}$ are the electric field and the electric displacement in the thickness direction z respectively, $Y_{p}$ and $Y_{s}$ are elastic modulus of the piezoelectric layer and substructure respectively, $e_{31}$ is the piezoelectric constant, $\varepsilon_{33}$ is the permittivity of the piezoelectric layer, *h* and δ are thicknesses of the substructure and piezoelectric layer respectively, ξ is the distance from the interface to the neutral surface of the composite beam which is derived by balancing the internal force of the cross section as $\xi =\frac{Y_{s}h^{2}-Y_{p}\delta^{2}}{2\left(Y_{p}\delta +Y_{s}h\right)}$ [9].

Integrating the moment of stress around the neutral surface of the composite beam for all the cross section and integrating the electric field and the electric displacement for all the volume of piezoelectric layer, leads to:

(2) $$\{\begin{array}{c} M\left(x\right)=\frac{K_{D}\varphi \left(x\right)}{\rho \left(x\right)}-e_{31}\left(\xi +\frac{\delta }{2}\right)\varphi \left(x\right)V\\ Q=e_{31}\left(\xi +\frac{\delta }{2}\right)\int_{0}^{L}\frac{1}{\rho \left(x\right)}\varphi \left(x\right)dx+\frac{\varepsilon_{33}S}{\delta }V \end{array}$$

where $M\left(x\right)=\int_{-\left(h_{S}-\xi \right)}^{\;\xi +\delta }T_{1}z\varphi \left(x\right)dz$ is the internal moment of a cross section of the beam, φ(x) is the width of the composite beam which is a function along the x-direction, $K_{D}=\int_{-\left(h_{S}-\xi \right)}^{\;\xi }Y_{S}z^{2}dz+\int_{\;\xi }^{\;\xi +\delta }Y_{P}z^{2}dz$ is the stiffness per width of the composite beam against bending, $V=E_{3}\delta$ is the voltage of the piezoelectric layer in the thickness direction *z*, $Q=\frac{1}{\delta }\int_{0}^{L}\int_{\;\xi }^{\;\xi +\delta }D_{3}dz\varphi \left(x\right)dx$ is the average charges on the surface of the piezoelectric layer, *S* is the surface area of piezoelectric layer.

**Figure 2:**
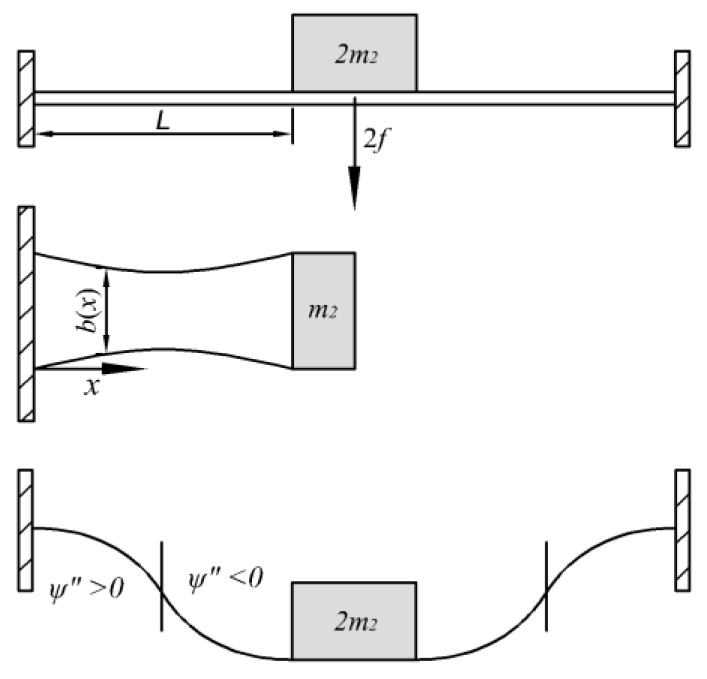
A double-clamped beam with a concentrated proof mass.

For analysis of the symmetric deformation of a double-clamped beam with a concentrated proof mass shown as in Figure 2, only a half part of both the beam and the mass needs to be considered.

The deflection can be written by:

(3) $$w\left(x,t\right)=\frac{\psi \left(x\right)}{\psi \left(L\right)}w_{m}\left(x,t\right)$$

where $w_{m}\left(t\right)=w\left(L,t\right)$ is the displacement of the proof mass and ψ(x) is the mode function of double-clamped beam with a concentrated proof mass. The bending curvature of the neutral surface can be expressed by $\frac{1}{\rho \left(x\right)}=\frac{\psi^{\prime \prime }\left(x\right)}{\psi \left(L\right)}w_{m}\left(t\right)$.

Substituting it into Equation (2), multiplying the former one by $\psi^{\prime \prime }\left(x\right)$ and then integrating it along the length direction x, leads to:

(4) $$\{\begin{array}{c} \int_{0}^{L}M\left(x\right)\psi^{\prime \prime }\left(x\right)dx=\frac{K_{D}}{\psi \left(L\right)}\left\{\int_{0}^{L}\varphi \left(x\right){\left[\psi^{\prime \prime }\left(x\right)\right]}^{2}dx\right\}w_{m}-e_{31}\left(\xi +\frac{\delta }{2}\right)\left[\int_{0}^{L}\varphi \left(x\right)\psi^{\prime \prime }\left(x\right)dx\right]V\\ Q=e_{31}\left(\xi +\frac{\delta }{2}\right)\frac{1}{\psi \left(L\right)}\left[\int_{0}^{L}\psi^{\prime \prime }\left(x\right)\varphi \left(x\right)dx\right]w_{m}+\frac{\varepsilon_{33}S}{\delta }V \end{array}$$

where *L* is the half length of the beam.

The virtual work principle can be written by:

(5) $$f\delta w_{m}=\delta \int_{0}^{L}M(x)\frac{\partial^{2}w}{\partial x^{2}}dx=\frac{\delta w_{m}}{\psi (L)}\int_{0}^{L}M(x)\psi^{\prime \prime }(x)dx$$

where *f* is generalized force of the proof mass.

Substituting it into Equation (4), leads to:

(6) $$\{\begin{array}{c} f=\frac{K_{D}}{\psi^{2}\left(L\right)}\left\{\int_{0}^{L}\varphi \left(x\right){\left[\psi^{\prime \prime }\left(x\right)\right]}^{2}dx\right\}w_{m}-e_{31}\left(\xi +\frac{\delta }{2}\right)\frac{1}{\psi \left(L\right)}\left[\int_{0}^{L}\varphi \left(x\right)\psi^{\prime \prime }\left(x\right)dx\right]V\\ Q=e_{31}\left(\xi +\frac{\delta }{2}\right)\frac{1}{\psi \left(L\right)}\left[\int_{0}^{L}\psi^{\prime \prime }\left(x\right)\varphi \left(x\right)dx\right]w_{m}+\frac{\varepsilon_{33}S}{\delta }V \end{array}$$

In the dynamic case, there is:

(7) $$f=ma\left(t\right)-m{\ddot{w}}_{m}-c{\dot{w}}_{m}$$

where $m=m_{2}+\int_{0}^{L}\psi_{T}\psi_{T}\mu \varphi \left(x\right)dx$, *µ* is the mass per unit width and per unit length of the composite beam, *c* is damping efficient of the beam, a(t) is the exciting acceleration.

Substituting Equation (7) into Equation (6) and making a derivative of the second one with respect to time *t*, leads to:

(8) $$\{\begin{array}{c} m{\ddot{w}}_{m}+c{\dot{w}}_{m}+kw_{m}-\Theta V=ma\left(t\right)\\ I=\Theta {\dot{w}}_{m}+C_{p}\dot{V} \end{array}$$

where:

(9) $$k=\frac{K_{D}}{\psi^{2}\left(L\right)}\int_{0}^{L}\varphi \left(x\right){\left[\psi^{\prime \prime }\left(x\right)\right]}^{2}dx$$

(10) $$\Theta =e_{31}\left(\xi +\frac{\delta }{2}\right)\frac{1}{\psi \left(L\right)}\int_{0}^{L}\varphi \left(x\right)\psi^{\prime \prime }\left(x\right)dx$$

(11) $$C_{p}=\frac{\varepsilon_{33}S}{\delta }$$

where *I* is electric current, *k* is called the stiffness, Θ is called the converting factor (or coupling factor), $C_{p}$ is the capacitance.

The energy efficiency depends on the converting factor. If the electrode is fully covered on the piezoelectric layer to form one electrode pair, integrating this in Equation (10), leads to:

(12) $$\Theta =e_{31}\left(\xi +\frac{\delta }{2}\right)\frac{1}{\psi \left(L\right)}\left[-\varphi^{\prime }\left(L\right)\psi \left(L\right)+\int_{0}^{L}\varphi^{\prime \prime }\left(x\right)\psi \left(x\right)dx\right]$$

For the rectangular beam, $\varphi^{\prime }\left(x\right)=0$, $\varphi^{\prime \prime }\left(x\right)=0$, there is Θ=0. For a trapezoidal beam $\;\varphi^{\prime \prime }\left(x\right)=0$, there is $\Theta =-e_{31}\left(\xi +\frac{\delta }{2}\right)\varphi^{\prime }\left(L\right)\psi \left(L\right)$. It can be seen that most charges are cancelled out because the charge sign of the left part is opposite the right one that resulted from changing the sign of the curvature. This phenomenon does not occur on the cantilever beam reported in [10] because there is no problem caused by the sign changing of bending curvature.

**Figure 3:**
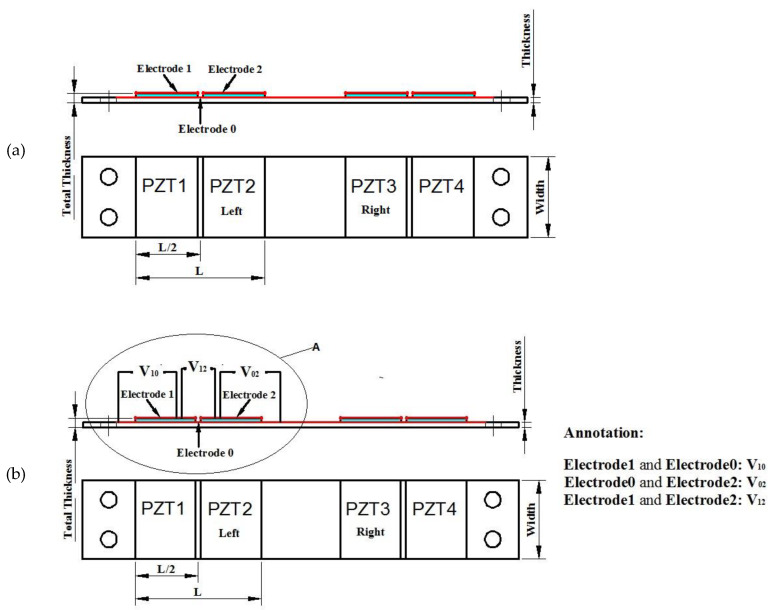

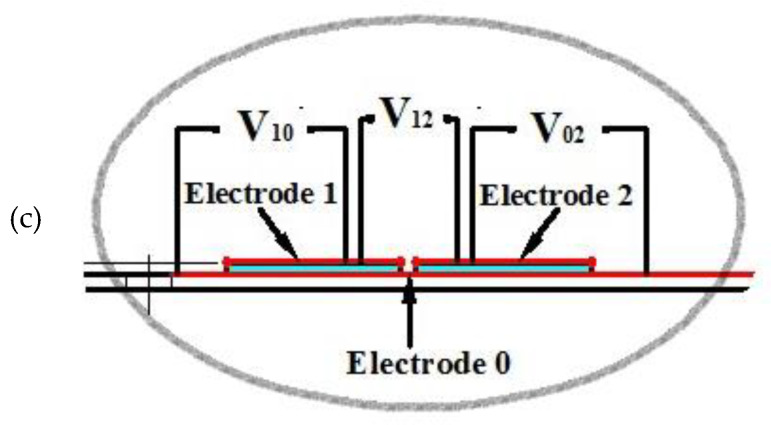
Double discrete electrodes and voltage outputs, (**a**) places of PZT and electrodes; (**b**)connections of electrodes for output voltages; (**c**) enlargement of the area A in (**b**).

To stack all the positive charges on one electrode and all the negative charges on the other electrode, the electrode on the upper surface should be divided into two parts and the lower surface can share one electrode. Through the positive and negative reverse connection $V_{12}=V_{10}-V_{20}$ shown as in Figure 3b, where the enlargement of the area A is shown as in Figure 3c, the positive and negative charges cannot cancel out each other so that more charges can be effectively collected.

In this case, the converting factor should be expressed as:

(13) $$\Theta =e_{31}\left(\xi +\frac{\delta }{2}\right)\frac{1}{\psi \left(L\right)}\left[\int_{0}^{\frac{L}{2}}\varphi \left(x\right)\psi^{\prime \prime }\left(x\right)dx-\int_{\frac{L}{2}}^{L}\varphi \left(x\right)\psi^{\prime \prime }\left(x\right)dx\right]$$

The energy efficiency depends on the converting factor. From its expression, it can be seen that, converting factor Θ depends on the width function φ(x). For different width shapes, there are different converting factors. To obtain a maximum converting factor, the width shape has to be optimized. For the symmetrical shape to the center point $x=\frac{L}{2}$ of the half beam, the above equation of the converting factor can be further derived as:

(14) $$\Theta =e_{31}\left(\xi +\frac{\delta }{2}\right)\frac{1}{\psi \left(L\right)}\left[2\varphi \left(\frac{L}{2}\right)\psi^{\prime }\left(\frac{L}{2}\right)+\varphi^{\prime }\left(L\right)\psi \left(L\right)+\int_{0}^{\frac{L}{2}}\varphi^{\prime \prime }\left(x\right)\psi \left(x\right)dx-\int_{\frac{L}{2}}^{L}\varphi^{\prime \prime }\left(x\right)\psi \left(x\right)dx\right]$$

**Figure 4:**
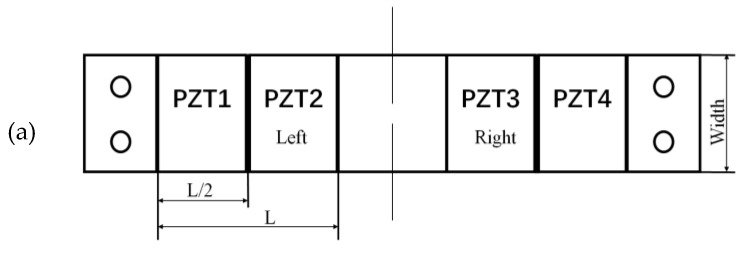

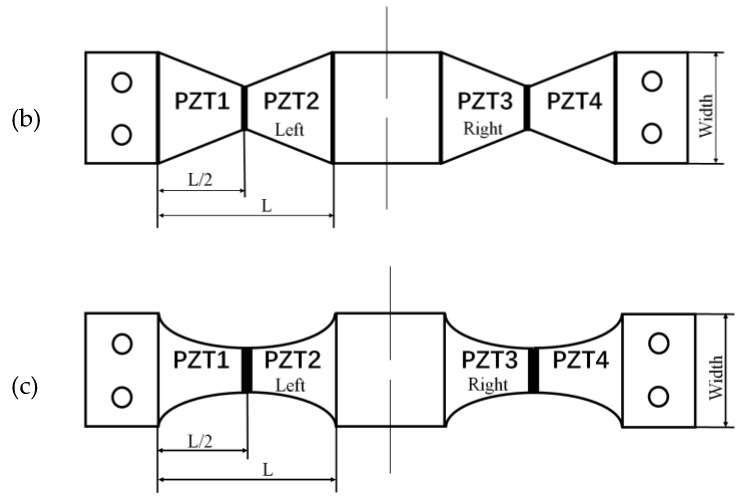
Three kinds of shapes. (**a**) Rectangular. (**b**) Trapezoidal. (**c**) Concave parabolic.

For a beam with double rectangular parts shown in Figure 4a, $\varphi^{\prime }\left(x\right)=0$ and $\varphi^{\prime \prime }\left(x\right)=0$, there is:

(15) $$\Theta =e_{31}\left(\xi +\frac{\delta }{2}\right)\frac{2}{\psi \left(L\right)}\varphi \left(\frac{L}{2}\right){\psi_{1}}^{\prime }\left(\frac{L}{2}\right)$$

For a beam with double trapezoidal parts shown in Figure 4b, $\varphi^{\prime \prime }\left(x\right)=0$, there is:

(16) $$\Theta =e_{31}\left(\xi +\frac{\delta }{2}\right)\frac{1}{\psi \left(L\right)}\left[2\varphi \left(\frac{L}{2}\right)\psi^{\prime }\left(\frac{L}{2}\right)+\varphi^{\prime }\left(L\right)\psi \left(L\right)\right]$$

For a beam with concave parabolic shape shown in Figure 4c, $\varphi^{\prime \prime }\left(x\right)$ is a constant, and there is:

(17) $$\Theta =e_{31}\left(\xi +\frac{\delta }{2}\right)\frac{1}{\psi \left(L\right)}\left\{2\varphi \left(\frac{L}{2}\right)\psi^{\prime }\left(\frac{L}{2}\right)+\varphi^{\prime }\left(L\right)\psi \left(L\right)+\varphi^{\prime \prime }\left[\int_{0}^{\frac{L}{2}}\psi \left(x\right)dx-\int_{\frac{L}{2}}^{L}\psi \left(x\right)dx\right]\right\}$$

From calculation of the converting factors corresponding to the above three shapes with the same width at the clamped end, it can be seen that, the double trapezoidal shape is better than the rectangular, whereas the concave parabolic shape is better than the double trapezoidal.

For an open circuit where there is no electric current, there is:

(18) $$V_{12}=\frac{\Theta }{C_{p}}$$

Substituting it into the first governing equation mentioned above, leads to:

(19) $$m{\ddot{w}}_{m}+c{\dot{w}}_{m}+\left(k+\frac{\Theta^{2}}{C_{p}}\right)w_{m}=ma\left(t\right)$$

If a(t)=Asinωt where $\omega^{2}=\frac{1}{m}\left(k+\frac{\Theta^{2}}{C_{p}}\right)$, the peak value of open voltage is:

(20) $${\bar{V}}_{12}=\frac{m\Theta }{2\zeta \left(kC_{p}+\Theta^{2}\right)}A$$

where ζ is damping ratio.

From this equation it can be seen that the equivalent stiffness $k^{*}=k+\frac{\Theta^{2}}{C_{p}}$ will be affected by the converting factor.

By solving this, when considering the coupling electric field, the open voltage can be obtained.

For a connected closed-circuit system with a load *R*, the circuit current can be expressed by $I\left(t\right)=\frac{V\left(t\right)}{R}$. The above equations can be rewritten as:

(21) $$\{\begin{array}{c} m{\ddot{w}}_{m}+c{\dot{w}}_{m}+kw_{m}-\Theta V=ma\left(t\right)\\ \Theta {\dot{w}}_{m}+C_{p}\dot{V}-\frac{V}{R}=0 \end{array}$$

In order to analyze the influence of the beam width, we can obtain a simplified quasi-static model by ignoring the inertial part and the damping part of the Equation (8) as:

(22) $$\{\begin{array}{c} kw_{m}-\Theta V=ma\left(t\right)\\ Q=\Theta w_{m}+C_{p}V \end{array}$$

In this case, the beam is just an elastic element in the vibration system. The charges are obtained as:

(23) $$Q=\frac{\Theta }{k}ma\left(t\right)+\left(\frac{\Theta^{2}}{k}+C_{p}\right)V$$

where $k=\frac{K_{D}}{\psi^{2}\left(L\right)}\int_{0}^{L}\varphi \left(x\right){\left[\psi^{\prime \prime }\left(x\right)\right]}^{2}dx$, $\Theta =e_{31}\left(\xi +\frac{\delta }{2}\right)\frac{1}{\psi \left(L\right)}\int_{0}^{L}\varphi \left(x\right)\psi^{\prime \prime }\left(x\right)dx$, $C_{p}=\frac{\varepsilon_{33}S}{\delta }$.

Taking the rectangular shape as an example φ(x)=constant, For an open circuit, there are:

(24) $$Q=\frac{e_{31}\left(\xi +\frac{\delta }{2}\right)\frac{1}{\psi \left(L\right)}\int_{0}^{L}\psi^{\prime \prime }\left(x\right)dx}{\frac{K_{D}}{\psi^{2}\left(L\right)}\int_{0}^{L}{\left[\psi^{\prime \prime }\left(x\right)\right]}^{2}dx}ma\left(t\right)$$

(25) $$V=\frac{\Theta }{C_{p}}w_{m}$$

It can be seen roughly that, as the width of the beam becomes narrower, the converting factor and the stiffness of the beam become smaller simultaneously. From the formula, the amount of charge seems to be independent of the width of the beam. The voltage is different. Although the converting factor and the capacitance become smaller simultaneously, the vibration amplitude of the mass will increase so that the voltage will increase. However, this is at the cost of increasing space.

From the perspective of the electrode coverage area, the simplified quasi-static model shows that when the electrode only covers the high-stress area at the root of the beam, whose length is $L^{\prime }$($L^{\prime }\ll L$), the converting factor and the capacitance will become small as $\Theta =e_{31}\left(e+\frac{\delta }{2}\right)\frac{1}{\psi \left(L\right)}\int_{0}^{L^{\prime }}\varphi \left(x\right)\psi^{\prime \prime }\left(x\right)dx$ and $C_{p}=\frac{\varepsilon_{33}SL^{\prime }}{\delta L}$ respectively due to the shorting of the integration area. Therefore, the voltage change is not too large. On the contrary, since the stiffness does not change, whereas the converting factor will become smaller due to the shorter integration region, and then the amount of charge will be reduced a lot.

Of course, the above discussion is just a rough analysis.

## 3. Experiments and Verifications

In order to verify the theoretical model, we conducted two kinds of experiments. One kind was for millimeter-scale structures, the other was for micro-scale structures.

The experimental setup includes a vibrating shaker controlled by a signal generator and a power amplifier in which alternative frequency and amplitude excitations can be provided, a dynamic signal analyzer was used to record output voltage, and an accelerometer was used to record the vibration acceleration of the shaker.

The open voltage outputs are recorded by a dynamic signal analyzer. Its internal resistance is 2 MΩ. This resistance is much higher than the impedance of harvester which is only about 10–20 kΩ.

**Figure 5:**
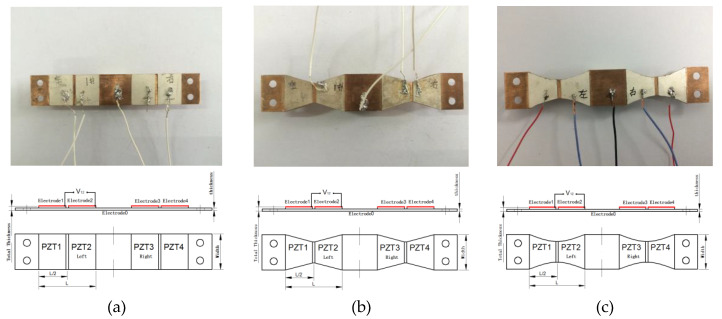
Three shape kinds of designed and manufactured double-clamped beam structures. (**a**) Rectangular, (**b**) Trapezoidal, (**c**) Concave parabolic.

For millimeter-scale structures, three shape kinds of double-clamped beam structures were designed and manufactured shown in Figure 5. One is rectangular as shown in Figure 5a, one is segmentally trapezoidal as shown in Figure 5b, and the other is concave parabolic as shown in Figure 5c. Two discrete pieces of polarized PZT-5H piezoelectric layer stick symmetrically on the upper surface of each half substructure beam. The substructure is made of copper material, whose Young’s Modulus is relatively small to benefit from the lower frequency. A concentrated proof mass is fixed at the center of the double-clamped beam. Corresponding discrete silver layers as electrodes are covered on the upper surface of the PZT-5H piezoelectric layers. A continuous silver layer as a sharing electrode is fully covered on the lower surface of the piezoelectric layer of each half beam to conduct the two piezoelectric parts of a half beam structure.

The open voltage $V_{12}$ is the voltage between the upper electrode 1 and electrode 2 corresponding to piezoelectric part 1 and piezoelectric part 2 by series connecting these two parts.

The material parameters of the substructure beam and the piezoelectric layer are listed in Table 1 where $\rho_{s}$ and are mass densities of the substructure beam and piezoelectric layer, respectively. The geometric parameters of the harvester structure are listed in Table 2 where ζ is damping ratio, *A* is the amplitude of the excitation acceleration, *L* is the half length of all these three kinds of beam, *a* is the base width of all these three kinds of beam, $b_{\mathrm{rect}}$, $b_{\mathrm{trapez}}$, $b_{\mathrm{parab}}$ are widths at center ($x=\frac{L}{2}$) of the half beam corresponding to the rectangular, trapezoid, and parabolic shapes, respectively.

**Figure 6:**
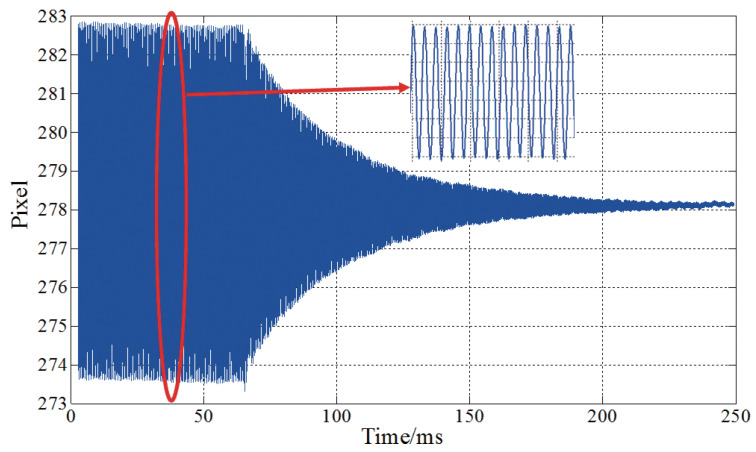
Measured amplitudes after excitation suddenly terminates.

The damping ratio ζ is obtained by testing based on the principles of vibration mechanics [19]. When the excitation of the beam structure in the resonance state suddenly terminates, the vibration amplitude of the structure will attenuate as a logarithm function. This amplitude (pixel values) is measured by high-speed camera (Photron SA4) as shown in Figure 6. For n cycles apart, logarithmic decrement of amplitude obeys the following relationship $\frac{1}{n}ln\frac{A_{i}}{A_{i+n}}=\frac{2\pi \zeta }{\sqrt{1-\zeta^{2}}}$. By use of the measured data $A_{i}$ and $A_{i+n}$, the damping ratio ζ can be calculated as 0.013.

**Figure 7:**
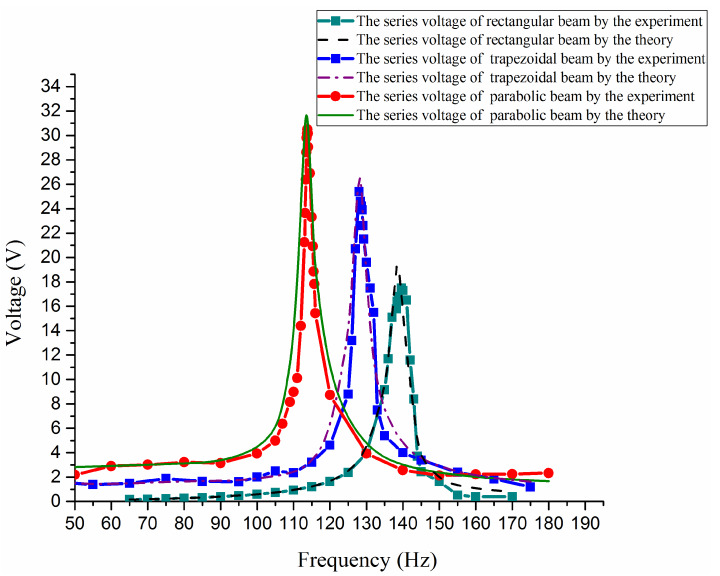
Open circuit voltage outputs versus exciting frequencies.

For these three different shapes, by experimental and theoretical analysis, some open voltage outputs are obtained shown as in Figure 7. From the curves it can be found that the voltage amplitude of the concave parabolic shape is the maximum and its resonant frequency is minimum among these three kinds of shape, whereas the rectangular shape has a minimum voltage amplitude and maximum resonant frequency.

**Figure 8:**
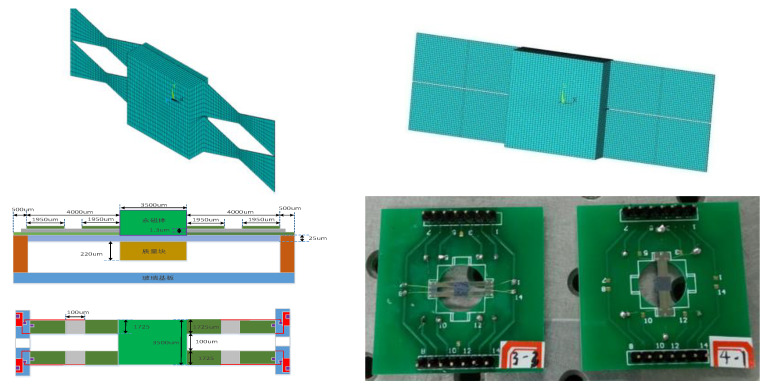
Double beams MEMS structures.

**Figure 9:**
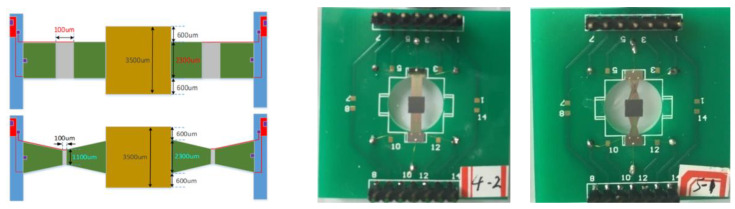
Single beam microelectromechanical system (MEMS) structures.

For micro-scale (MEMS) structures, two shape kinds of double-clamped beam structures were designed and fabricated with the MEMS process [20]. One kind of structure is a double-beam structure as shown in Figure 8, the other is single beam structure, as shown in Figure 9. Every kind of structure includes two kinds of beam shapes. One kind is a rectangular shape, the other is a concave trapezoidal shape. They are all fabricated with the MEMS process. The MEMS process includes: (a) the oxidizing of the silicon wafer into SiO2 and the patterning of the SiO2 layer; (b) the sputtering of the Ti layer and Pt layer; (c) depositing of the PZT thin film on the Pt/Ti/SiO2/Si substrate with the sol-gel method; (d) the fabricating of the top electrode with the sputtering method; (e) the corroding of the PZT thin film; (f) the freeing of beam structures by a dry-etching process.

Because the micro-scale structure is fabricated by MEMS processing technology, the base beam (substructure) is made of silicon (Si) material which is compatible with this process.

The thickness of the substructure beam is 27.7 µm, its Young’s modulus is 190 GPa, and its mass density is 2330 kg/m^3^. The piezoelectric layer is PZT whose thickness is 1.3 µm, Young’s modulus is 60 GPa, mass density is 7720 kg/m^3^. The lengths of a half beam for both double beam and single beam are 4000 µm, the width at the fixed end of the single beam for both rectangular and trapezoid shapes is 2300 µm, and the width at the fixed end of the double beam is 1725 µm. Other geometric parameters are shown in Figure 9.

**Figure 10:**
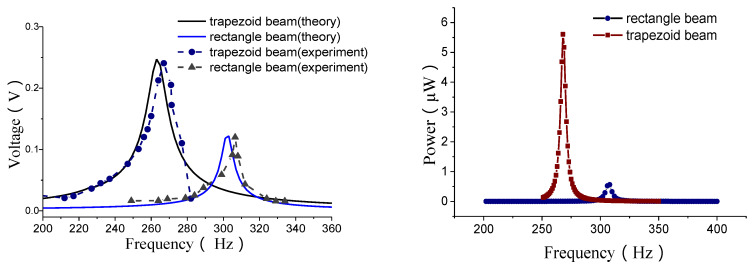
Open voltage and power outputs.

The open voltage and power outputs for a double-beam structure are shown in Figure 10.

**Figure 11:**
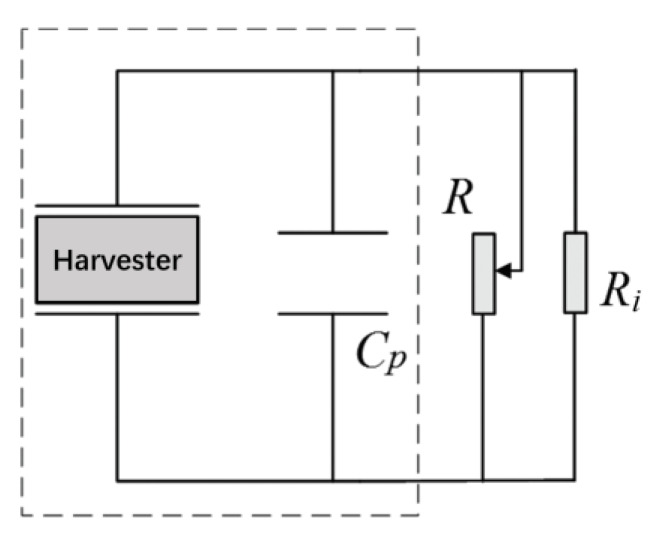
A loop circuit with an adjustable load resistance.

**Figure 12:**
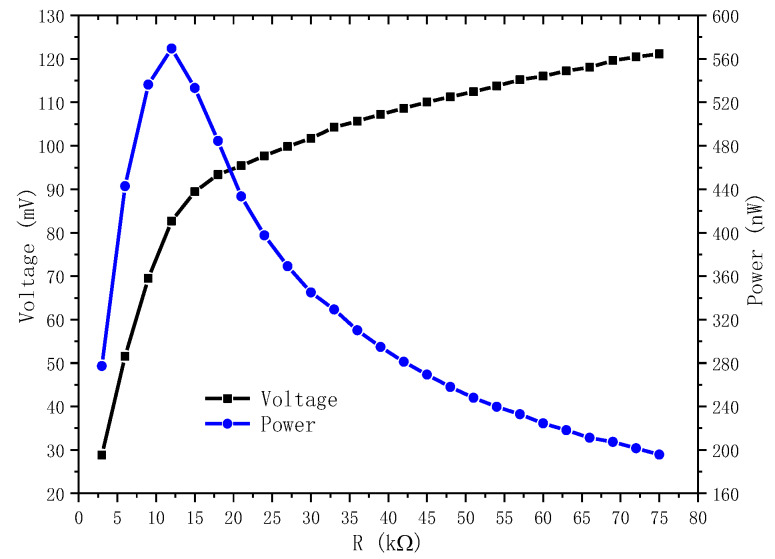
Curves of voltage and power.

The power is calculated according to the optimal load resistance. The optimal load resistance is obtained through testing. The test is conducted for a loop as shown in Figure 11, where the load resistance is adjustable. A dynamic signal analyzer is used to test the voltage across the load resistance. The internal resistance $R_{i}$ of the dynamic signal analyzer is very high, which is 2 MΩ, the load resistance *R*, the resistance of the energy harvester and $R_{i}$ form a parallel connection. For the resonant frequency, the voltage will increase as the adjustable resistance increases to the open circuit voltage, as shown in Figure 12. At the beginning it increased very quickly. The output power can be calculated according to the voltage and adjustable load resistance by $p=\frac{V^{2}}{R}$. In the first stage, the output power increases with the increase of the load resistance. But after a peak value, it will decrease. The resistance corresponding the peak value is 12 kΩ which is called the optimal load resistance. This peak value of power is the power for the given frequency we obtain. In the same way, a series of power corresponding different frequencies as shown in Figure 10 can also obtained.

**Figure 13:**
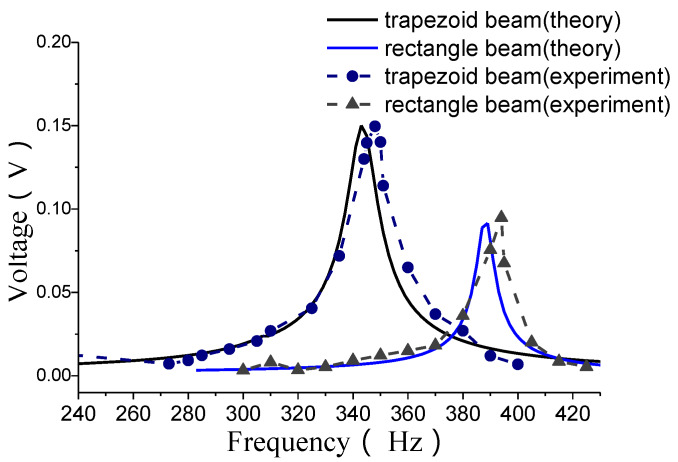
Open voltage outputs.

The open voltage outputs for single beam structure are shown in Figure 13.

From these results, it can also be observed that, the energy outputs of a MEMS harvester with a trapezoidal-shaped beam are better than that with a rectangular-shaped beam. At the same time, the resonant frequencies will decrease.

## 4. Conclusions

In this work, two kinds of double-clamped piezoelectric energy harvester with different width shapes are investigated. In an experimental and analytical way, some electrical outputs are obtained. Not only for millimeter-scale structures but also for micro-scale (MEMS) structures, the theoretical results are in good agreement with those of the experiments. From these results, it can be observed that both the width shapes of the beams and the arrangement of the electrodes have a direct effect on the electrical outputs of piezoelectric energy harvesters.

In future research, on the one hand, through MEMS process design and the processing, we hope to complete the analysis of the micro-scale parabolic beam structure; on the other hand, we hope to explore the effects of thickness shape (such as wedge, tapering, etc.) on the electric output of the energy harvester.

## Figures and Tables

**Table 1:** Material parameters.

Parameters	Value
$\rho_{s}$	8300 kg/m^3^
$\rho_{p}$	7450 kg/m^3^
$Y_{s}$	131 GPa (10^9^ N/m^2^)
$Y_{p}$	60 GPa (10^9^ N/m^2^)
$\mu_{s}$	0.35
$\varepsilon_{33}$	1470 $\varepsilon_{0}$
$\varepsilon_{0}$	8.854 × 10^−12^ F/m
$e_{31}$	11.16 C/m^2^

**Table 2:** Geometric parameters.

Parameters	Value
*h*	0.3 mm
δ	0.2 mm
2*m*_2_	17 g
ζ	0.013
*A*	2 m/s^2^
*L*	23 mm
*a*	15 mm
$b_{\mathrm{rect}}$	15 mm
$b_{\mathrm{trapez}}$	12 mm
$b_{\mathrm{parab}}$	12 mm
